# Multi-Stakeholder Perspectives of Factors That Influence Contact Centre Call Agents’ Workplace Physical Activity and Sedentary Behaviour

**DOI:** 10.3390/ijerph15071484

**Published:** 2018-07-13

**Authors:** Abigail Morris, Rebecca Murphy, Sam Shepherd, Lee Graves

**Affiliations:** Research Institute for Sport and Exercise Sciences, Liverpool John Moores University, Liverpool L3 3AF, UK; r.c.murphy@ljmu.ac.uk (R.M.); s.shepherd@ljmu.ac.uk (S.S.); l.e.graves@ljmu.ac.uk (L.G.)

**Keywords:** sedentary behaviour, physical activity, workplace, intervention development

## Abstract

Contact centre call agents are highly sedentary at work, which can negatively affect cardio-metabolic health. This qualitative cross-sectional study explored factors influencing call agents’ workplace physical activity (PA) and sedentary behaviour (SB), and perspectives on strategies to help agents move more and sit less at work. Semi-structured interviews and focus groups with call agents (*n* = 20), team leaders (*n* = 11) and senior staff (*n* = 12) across four contact centres were guided by the socio-ecological model and analysed thematically. Agents offered insights into the impact of high occupational sitting and low PA on their physical and mental health, and factors influencing their motivation to move more and sit less at work. Team leaders, although pivotal in influencing behaviours, identified their own workload, and agents’ requirement to meet targets, as factors influencing their ability to promote agents to move more and sit less at work. Further, senior team leaders offered a broad organisational perspective on influential factors, including business needs and the importance of return on investment from PA and SB interventions. Unique factors, including continuous monitoring of productivity metrics and personal time, a physical connection to their workstation, and low autonomy over their working practices, seemed to limit call agents’ opportunity to move more and sit less at work. Proposed strategies included acknowledgement of PA and SB within policy and job roles, height-adjustable workstations, education and training sessions and greater interpersonal support. Additionally, measuring the impact of interventions was perceived to be key for developing a business case and enhancing organisational buy-in. Multi-level interventions embedded into current working practices appear important for the multiple stakeholders, while addressing concerns regarding productivity.

## 1. Introduction

High levels of sedentary behaviour (SB), defined as “any waking behaviour characterized by an energy expenditure ≤1.5 METs, while in a sitting, lying or reclining posture” [1], are associated with distinct metabolic and physiological processes [2,3]. These processes increase the risk of developing cardiovascular disease, type 2 diabetes, some cancers and premature mortality, independent of physical activity (PA) levels [3,4,5]. Frequent breaks to prolonged sitting are also associated with favourable cardio-metabolic profiles [6,7]. Accordingly, further to public health guidelines to accrue 150 min of moderate or 75 min of vigorous intensity PA weekly, adults are recommended to reduce the time spent in total and prolonged periods of SB [8].

Adults engage in SB across various domains including transport, leisure and occupation [9,10,11]; however, occupational sitting contributes up to two thirds of office workers’ total daily sedentary time [12,13]. Further, adults in mainly desk-based occupations are exposed to forced periods of inactivity and static seated postures, which are ergonomic hazards in the physical work environment [3]. Workplaces that employ mainly desk-based workers are therefore vital settings to prevent exposure to such hazards, via targeted reductions in total and prolonged SB, and PA promotion [3,14,15,16]. This is supported by recommendations for mainly desk-based workers to accumulate 2–4 h per day of standing and light activity during working hours, with emphasis on regularly breaking up seated work [7].

Multiple factors contribute to high levels of SB and low PA at work in traditional office workers, including high workload and social norms surrounding sedentary working behaviours [17,18,19]. Accordingly, multi-level interventions targeting several such factors have proven effective at reducing total [20] and prolonged [21] sitting time at work in traditional office workers. Few trials, however, have succeeded in promoting PA at work [22], and fewer trials have reported long-term positive behaviour change [21]. Further, little research has sought to promote PA and/or reduce SB in the contact centre setting.

In the UK, ~4% of the working age population (~766,000 adults) are employed as call agents in contact centres [21]. The work of call agents in contact centres is typically monotonous [23] and characterised by a high volume of customer service interactions that require telephone and computer assistance [24]. Call agents report significantly lower job satisfaction and higher job-related anxiety and depression compared to workers in clerical, secretarial, technical support and professional jobs [23]. Furthermore, compared to traditional office workers, call agents have less perceived autonomy over their working tasks [23], and spend more time at work sedentary and desk-based (90% vs. 77%) [25,26,27], often in periods of ≥30 min bouts [26]. This suggests that call agents are at greater risk for non-communicable disease than traditional office workers. The distinct nature of contact centre work therefore suggests that the factors influencing call agents’ workplace PA and SB may differ from traditional office workers.

Currently, there is limited awareness of the factors that influence PA and SB at work in highly sedentary contact centre call agents. Contact centre managers suggest that improved employee wellbeing and productivity are key drivers for organisational investment in health and PA strategies; however, they perceived agents’ time away from calls as a barrier to daily PA promotion due to potential productivity losses [28]. A recent trial compared objective productivity metrics (considered as the number of successful calls taken per hour) between seated controls and a stand-biased or height-adjustable workstation intervention arm across a 6-month intervention. Intervention agents averaged 0.5 more successful calls per hour compared to their seated counterparts, despite shorter tenure in their current role [29]. This trial suggests that height-adjustable workstations allow agents to maintain and potentially improve their performance over time, which appears important for managers. However, there is a lack of robust evidence demonstrating the effectiveness of height-adjustable workstations for reducing total and prolonged SB in call agents. Furthermore, development of multi-level trials in traditional office workers suggest targeting influential factors across organisational, environmental, interpersonal and intrapersonal levels, as outlined by the socio-ecological model (SEM), are more effective than single level interventions alone [10,30,31].

To date, only two trials have investigated the effect of a multi-level intervention on call agents sitting time at work. The “Opt to Stand” quasi-experimental trial compared the provision of height-adjustable workstations, daily emails and brief training on workday sitting and PA, to a control group [32]. Due to poor compliance to objective monitoring, participants self-reported data was used to identify a reduction in sitting time (−64, −76, −100 min/workday) and increased standing (73, 96, 51 min/workday) compared to controls at weeks 1, 4 and 19, respectively. While positive, the short-term follow-up and subjective assessment of sitting and standing limit knowledge of the effectiveness and sustainability of this multi-level intervention [32]. Furthermore, following the “Sit Less Move More” intervention, consisting of emails, posters and timer lights to prompt sitting reduction among emergency call agents with individual height-adjustable workstations, call agents identified high levels of fatigue and high work pressures, as barriers to sitting less during the 11-week intervention [33]. Although no effectiveness data has been published, these findings provide useful fidelity and contextual considerations for future interventions in this setting [34].

In accordance with the limited evidence in this setting, no published research has reported the use of formative work to determine the specific factors influencing call agents SB and PA prior to the development of targeted intervention strategies. This approach is supported by the Medical Research Council (MRC) framework [35], which advocates formative work as a key phase of intervention development prior to piloting, evaluation and implementation, to enhance the effectiveness and sustainability of tailored intervention strategies. In line with the MRC framework, therefore, and underpinned by the SEM [10], the primary aim of this study was to explore the factors influencing call agents’ workplace PA and SB, from the perspective of call agents, their team leaders, and senior staff. The secondary aim was to identify strategies that may help agents to move more and sit less at work, to inform future intervention development in this unique setting.

## 2. Methods

### 2.1. Study Design

This qualitative cross-sectional study used focus groups and interviews to explore contact centre stakeholder perspectives. Questionnaires were completed after the focus group or interview to describe the sample. Qualitative findings are reported in line with the consolidated criteria for reporting qualitative research (COREQ) checklist [36]. Ethical approval was obtained from Liverpool John Moores University research ethics committee (16/SPS/033).

### 2.2. Participants and Settings

A convenience sample of participants were recruited from four private contact centre companies with branches in the North West of England, situated in both urban (*n* = 2) and rural (*n* = 2) settings. Two of the four centres implemented height-adjustable workstations and frequent break schedules as remedial measures for employees with musculoskeletal or chronic medical conditions, following a display screen equipment assessment [37]. Each company expressed interest in the study following an invited presentation by the research team at a North West of England contact centre forum. After individual meetings to discuss the study aims and requirements, each company provided gatekeeper consent to recruit their employees and conduct the research in their centre. Call agents, team leaders and senior staff (consisting of human resources, head of centre, support team and engagement manager roles) received a recruitment message and participant information sheet, developed by the research team, via each company’s internal e-mail system. Employees were eligible if they were ≥18 years old and ≥0.8 full time or part time equivalent worker in a call agent, team leader or senior staff role. All call agents operated in inbound call taking roles. Participants had 2 weeks to express interest by contacting the main author via email or an assigned contact within each centre. Interested and eligible participants (call agents *n* = 20, team leaders *n* = 11, senior staff *n* = 12) provided written informed consent.

### 2.3. Focus Groups and Interviews

Focus groups were used to elicit in-depth insights across the three key stakeholder groups within four contact centres. Focus groups have similarly been used to explore employee and employer attitudes towards potential SB and PA intervention strategies [19], and employee perceptions of a SB workplace intervention [38]. Compared to quantitative methods such as surveys, focus groups can highlight attitudes and assumptions within a population, with participant interactions able to explore the extent to which perspectives are consistent and/or contrary [39]. The aim was to recruit a minimum of 4–8 participants per focus group in line with previous recommendations [36,40]. However, due to the number of call agents who expressed interest, conflicting work schedules, and, staffing numbers for team leaders and senior staff, focus group size ranged between 2 and 6 participants, and three one-on-one interviews were conducted (two with team leaders and one with a senior staff member). Data collection took place on site at each company’s North West branch during working hours between July and October 2016. Accordingly, the first author, who is experienced in qualitative research, conducted six semi-structured focus groups with call agents, two with team leaders and four with senior staff. To increase homogeneity, participants were grouped according to job role to reflect the hierarchical organisational structure [41]. A team leader was present during two call agent focus groups in company 3; however, their contributions were considered important to the research objectives and did not appear to impact the agents’ willingness to openly discuss their perspectives and experiences [40]. To maintain participant confidentiality and safeguard participants and the researcher, data collection occurred in a meeting space familiar to all participants that could be overseen, but not overheard. All focus groups and interviews were recorded using a digital audio recorder and ranged from 29 to 87 min in duration (mean 56 ± 14 min).

To enhance the credibility and trustworthiness of the data, the focus group schedule was developed in line with organisational, environmental, interpersonal and intrapersonal levels of the SEM [10,30], and grounded within current literature advocating a multi-level approach to workplace intervention development [21,42]. The protocol for delivering the focus groups and interviews was standardised, using the semi-structured schedule as a guide to promote a commonality throughout the focus groups. To allow participants to respond openly and freely however, flexibility in the order and sequence of questions was permitted [40]. Questions addressed perceived factors influencing call agents’ workplace PA and SB. Within this, discussion areas included current working practices and initiatives that promote or negate SB and PA, perceived roles and responsibilities for promoting health and wellbeing, and employee perspectives on the current workplace recommendations [43] (see Supplementary Materials). The first author developed the focus group schedule, which was reviewed by two members of the research team (LG, RM) during team briefing sessions. No pilot interviews or focus groups were conducted prior to data collection, although consistent themes emerged across the 12 focus groups and 3 interviews, which suggests saturation was reached [36].

### 2.4. Focus Groups and Interviews Analysis and Representation

Focus groups and interviews were transcribed verbatim, with participants anonymised during transcription. A thematic approach was adopted, which allowed the flexibility to identify themes across the complete data set, in relation to the factors influencing agents workplace PA and SB [44,45]. Analysis began concurrently with data collection through a reflective commentary, which contained the researcher’s initial thoughts and emerging patterns in the early stages of analysis [41,46]. The SEM provided the point of departure framework for the deductive analysis. During the inductive process, transcriptions were read and re-read to familiarise the researcher with the complete data set, and initial codes were generated from a piece of text that related to factors influencing workplace SB and PA [46], prior to being imported into QSR NVivo software 10 package. Higher-order themes were generated from emerging patterns within the initial coded data, and further grouped into levels within the SEM. Sub-themes provide a structure to complex higher-order themes by adding a rich context to the research question beyond the pre-defined categories of the SEM [45]. At this stage the coding framework was presented by the first author and reviewed by all authors during a debriefing session that allowed refinement and further defining of emerging themes [46], with this triangulation adding credibility and trustworthiness to the analysis process [41].

### 2.5. Surveys and Questionnaires

To describe the sample characteristics, participants completed a non-validated survey, adapted from a previous workplace intervention [47]. Participants self-reported their age, gender, ethnicity, marital status and education level (sociodemographic factors), employment history, employment status, job category, hours worked per week and main work tasks (work history), the number of people in their office (work environment), and, occupational transportation mode. The International Physical Activity Questionnaire (IPAQ) (long form) assessed habitual PA over the previous 7-days [48]. Twenty-seven questions assessed the frequency and duration of moderate and vigorous-intensity PA and walking activities undertaken across four domains (occupational, transport, household and recreation). Total PA scores were calculated through summation of duration and frequency of each activity across each domain, and classified as low (<600 MET minutes·week), moderate (600–2999 MET minutes·week) or high activity (≥3000 MET minutes·week). Estimated weekend and weekday sitting time values are not included in the total PA score. Previous studies have shown good test-retest reliability (0.8), with fair-to-moderate criterion validity between the IPAQ and accelerometer data (0.33, 95% CI 0.26–0.39) [48].

The Workforce Sitting Questionnaire assessed previous 7-day workday and non-workday sitting during travel, work, watching TV, using a computer (at home) and during other leisure activities [49]. Total sitting time for a typical workday and non-workday were calculated by summing sitting times across each domain. Participants reported the number of workdays during the previous 7-days, which allowed a weighted mean for total sitting time per day to be calculated. The questionnaire has good-to-excellent test-retest reliability (0.65–0.80) [49]. Lastly, participants completed a 12-item survey to measure functional health and wellbeing (SF12v2) [50] across 8 health domains. The standard scoring algorithm aggregated domain scores into higher-order sub-scales of a physical (PCS, %) and mental component summary (MCS, %). Mean scores below 50% indicated a below average physical or mental health status [51]. PCS and MCS have displayed high internal consistency (α > 0.80) and are widely used to provide an overview of health status [50].

## 3. Results

The sample was predominantly comprised of female, White British, single, and full-time employees (Table 1). Agents predominately had ≤3 years tenure in their current role. All participants reported their daily working tasks were mainly seated, although call agents reported a higher volume of occupational sitting time than team leaders and senior team members. Self-reported previous 7-days PA ranged between 460 and 925 MET min·week for agents, 537–1190 MET min·week for senior team leaders and 150–4690 MET min·week for team leaders, with team leaders typically reporting the highest PA levels. Team leaders and senior team members reported average physical health scores, while agents reported below-average physical health scores. Both team leaders and call agents reported lower than average mental health scores according to the U.S. mean (50 ± 10%), with scores lowest for team leaders [51,52].

**Figure 1:**
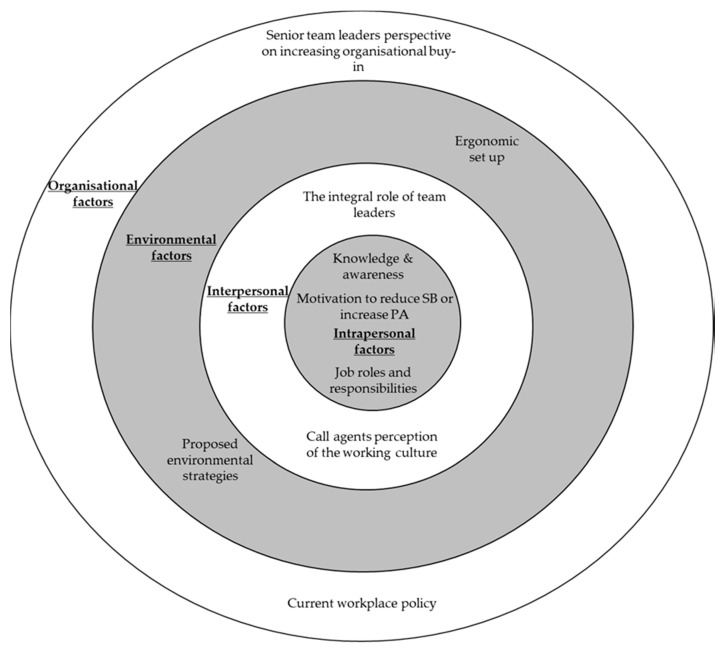
An overview of the higher-order themes that represent factors influencing call agents’ workplace PA and SB across four levels of the SEM.

Factors from all levels of the SEM were perceived to influence call agents’ PA and SB at work. Higher-order and sub-themes are presented in accordance with levels of the SEM and schematically (Figure 1). Throughout the results, data extracts are presented with participant number (e.g., P21), job role (AG = Agent, TL = Team leader, ST = Senior team), company number (1–4) and an indication of focus group or interview contribution (FG or I).

### 3.1. Intrapersonal Factors

#### 3.1.1. Knowledge and Awareness

Consistent with the Workforce Sitting Questionnaire data, agents were aware they spent the majority of a typical working day seated and desk-based. Call agents reported that minimal variation in their work tasks reduced the opportunities to break their SB and be active at work.

“*I’m sat down really. I don’t move, I really don’t move. So it’s completely sedentary until break about quarter past one and I’m sat down then as well.*”

P38 (AG4 FG)

Agents commonly acknowledged the presence of negative physical (weight gain and musculoskeletal discomfort) and psychological (low mood and low energy) effects following prolonged occupational and daily sitting.

“*I’ve put on weight, and my back kills sometimes, yes, from just having like really long days sat down, especially on the days that I don’t do anything after work as well. So like if I’m sat down all day at work, and then I go home and I’m sat in a chair at home or whatever when I go home, my back’s killing me.*”

P28 (AG4 FG)

Despite the sedentary nature of the job, agents perceived a number of benefits to moving more and sitting less, including feeling more alert on calls and less fatigued, improved general mood, wellbeing, social relationships and productivity, weight loss, and reduced musculoskeletal discomfort.

“*I’ve noticed that you kind of, when you’re up and about, you’re constantly moving and generally sort of feel a bit better by sort of moving and doing more things.* (…) *You may then get better working environments and more effective work from each individual if you do introduce more activity throughout the day.*”

P36 (AG4 FG)

Compared to agents and team leaders, the majority of senior team members were educated to a tertiary level. Education level however did not appear to influence knowledge and awareness of national PA guidelines [8] and recommendations for mainly desk-based workers [43], which was low to non-existent across agents, team leaders and senior team members.

“*Really? I didn’t know about that (workplace recommendations). I was just more thinking about it’s my eyes. I didn’t actually think me as like my body and everything.*”

P6 (AG1 FG)

In response to the workplace recommendations [43], most agents and team leaders felt that achieving a reduction of 2–4 h of total sitting was “*a hell of a lot of time*” (P32 TL3 FG) and even “*laughable*” (P8 AG1 FG) in the current working environment. Most agents were unsure how they could feasibly reduce or break up their sitting time to meet the recommendations.

“(Reducing sitting by 2–4 h per day) *That’s just not feasible working here, I don’t think. It would just be virtually impossible to cut* (sitting) *down by so much.*”

P6 (AG1 FG)

Accordingly, all stakeholder groups proposed further education and training as a strategy to promote knowledge and awareness of national guidelines and workplace recommendations for PA and SB, and additionally, the relationship between PA, SB and health.

“(I would) *just like more information on what the benefits are of doing it* (breaking sitting time), *be that health-wise or be that job-wise. Because obviously, at the moment you know realistically you shouldn’t be sat down all day, because it’s not good for you, but you don’t know the reasons, like what’s the benefits.*”

P1 (TL1 FG)

#### 3.1.2. Motivation to Reduce SB or Increase PA

When reflecting on factors influencing SB and PA at work, several call agents felt a lack of energy and feeling “*lethargic*” (P22 TL3 FG) reduced their daily motivation to break up sitting time, take active breaks or be more physically active during or after work.

“*It’s the fact that I think you just feel so drained and so tired from looking at the screen and just sitting there* (…) *and I just think you don’t feel motivated.*”

P4 (AG1 FG)

Several call agents identified sitting as an ingrained behaviour in their daily work routine, including during break times.

“*I would spend it* (break times) *at my desk, or if I think I’ve got enough time, I go into the little short room with the sofa. I go and lie on the sofa.*”

P33 (AG3 FG)

All agents reported high daily call volumes, and several reported frequent exposure to incivility during customer calls. These agents subsequently emphasised that a primary motive to break away from their workstations was to preserve their positive mental health rather than a desire to take an active break.

“*…so I tend to go in there, plug myself into my iPod, listen to a book, and so three quarters of an hour, just take myself away. I’m still sat. I don’t walk anywhere. But I kind of take myself away from work, and then come back mentally afterwards.*”

P15 (AG2 FG)

In contrast, some agents’ awareness and perception of their prolonged sitting motivated them to break away from their workstations during scheduled break times, or seek opportunities to break up sitting time during the day, to mitigate the negative effects of prolonged sitting.

“*I find with myself, when I’m sat down for too long, I tend to feel it more than anything, so I find that even if the opportunity to go up and do a brew run, say, getting myself moving, it tends to help kind of getting the blood flowing, shall we say, so when I do sit back down again and I’m back on my calls, it helps with keeping me alert, I think.*”

P34 (AG3 FG)

To promote awareness of, and increase agent motivation for future PA and SB strategies, agents suggested their organisations could use frequent verbal (during team meetings and one-to-ones), visual (posters, notice boards and flyers in communal areas) and electronic (via email, intranet or the Internet) promotional messages.

“*…it’s not really promoted on a daily basis. Because it’s the same with advertising. If you don’t advertise it, if you’re not going to notice it.*”

P34 (AG3 FG)

While agents reflected upon the factors influencing their daily motivation to move more and sit less, team leaders and senior team members contrastingly focused on previous uptake by agents to formal health and wellbeing initiatives, including running clubs, lunchtime walks, yoga, subsidised gym memberships and team competitions. Team leaders and senior team members often attributed poor uptake and limited longevity of initiatives to low call agent motivation, and perceived low motivation as a barrier influencing agents’ engagement in future workplace initiatives.

“*…the challenge they* (team leaders) *have is getting* (health and wellbeing initiatives) *off the ground. It’s not because of the effort we put into it, it’s because of the effort that the individual wants to give back. That’s the challenge.*”

P24 (ST3 I)

“*You might find that people will ask for something, even push for, but not use when it’s delivered and the opportunity’s there.*”

P34 (TL4 I)

Previous delivery of formal health and wellbeing initiatives across all centres was non-compulsory; therefore, agents were able to choose whether to engage or not.

“*We don’t want to be kind of dictating to people what they do and how they do it you know, poking people when they’ve been sat down for too long.*”

P18 (ST1 FG)

“*…well you can take the horse to water can’t you but you know there’s only so much you can, you can do, you can put it there, you can make it as, you know as available as possible but it then does rely on peoples sense of self responsibility to engage in that.*”

P11 (ST2 FG)

Accordingly, several senior team members emphasised the importance of understanding call agents’ wants and needs in relation to health and wellbeing, before developing future initiatives to promote PA and reduce SB in agents at work.

“(We need) *wellbeing interventions that are tailored to the specific wants and needs of our agents, and for that, we require agent feedback* (…) *we need to understand what the specific definition of wellbeing is for the individual to work for us in our contact centres* (…) *unless we get to that definition, then it’s likely that whatever we try and do,* (…), *we’ll miss the mark, because we’re not actually creating a solution that is fixing the problem, because the problem hasn’t been identified.*”

P26 (ST2 FG)

#### 3.1.3. Job Roles and Responsibilities

Agents, team leaders and senior team members acknowledged that in order to meet their job requirements and maintain productivity levels, agents need to be seated to take calls while simultaneously inputting information into the computer systems.

“*That’s the job, isn’t it? You’re employed to be sat down to take calls.*”

P34 (AG3 FG)

“*I just think, well, they’re paying me to do a job, so why should they pay me for doing my exercises.*”

P9 (AG1 FG)

Agents frequently reported a major barrier to sitting less at work was the potential negative impact on their productivity, which some felt could jeopardise their job security.

“*There’s no requirement within that role for us to get up and wander around. There’s nothing within the job description that says we need to do that.*”

P15 (AG2 FG)

“*I’ve not been here that long, to be honest, and I think that’s probably why I’m more anxious about security of the job.*”

P4 (AG1 FG)

Several agents expressed a unique desire for increased autonomy over their working practices, although interestingly, autonomy was not always perceived as a facilitator for moving more and sitting less.

“*I’d just like to be able to work standing up if I choose to, to have a desk that you can perhaps move up so that I can stand up for half an hour, because I’m fed up of sitting down.*”

P16 (AG2 FG)

“*It’s like me, I choose to sit down all day, I choose to sit in the bistro. It’s not the company making me do that, that’s just the way I am.*”

P15 (AG2 FG)

Encouragingly, call agents, team leaders and senior team members similarly supported the notion of agents becoming more active and less sedentary at work. Team leaders perceived that part of their role was to instigate PA, and SB reductions, in agents, and suggested their unique knowledge of their team’s dynamics and individual agents could help tailor PA and SB approaches to meet individual agents’ needs and preferences.

“*…I manage a team of people and I’ve got you know different types of people, some that are quite active, some that aren’t quite as active as each other.*”

P23 (TL3)

“*I think something that we could tailor to the individual needs as well.*”

P21 (TL3 FG)

Team leaders, however, perceived a high workload as a barrier to initiating and promoting PA and SB strategies at work. To that end, team leaders highlighted the need to consider the burden placed upon middle management in the design and implementation of future interventions.

“*…you’ve got a pile of escalations that have piled up, a pile of credits that have piled up, a load of emails that your line managers have sent you and they say ‘well why haven’t you done that?’* (…) *Sometimes you’re distracted, being a manger because of all the tasks that you have to do in a day, I don’t know how you find the balance to be honest.*”

P20 (TL3 FG)

To offset the burden placed upon team leaders, several team leaders and senior team members suggested implementing localised workplace champions.

“*You’ll get a fantastic management buy-in to those kind of theories, because it’s offloading a responsibility to a third party in a recognised format* (…) *somebody like a workplace champion would* (…) *decide to be involved, raise awareness* (…) *none of this would be for everybody, but to have, again, a workplace champion in place that understood, was a point of contact* (…) *I can see that working really quite well.*”

P43 (TL4 I)

Senior staff in company 1 recognised workplace champions as an opportunity to develop specific role requirements and provide professional development training for existing call agents.

“*…I think that’s definitely something that we can take away isn’t it as an idea.* (…) *is anybody interested* (…) *almost like applying for a role.*”

P18 (ST1 FG)

In addition, workplace champions were perceived as a strategy to shift attitudes surrounding workplace PA and SB, and, to promote sitting reductions and active breaks. At the time of data collection, company 2 used workplace champions to tackle mental health at work, with one team leader describing that it felt intuitive to include discussions of mental and physical health within their mental health champion role.

“*Most definitely yeah. I mean well that’s just part and parcel if it isn’t it that you know, in mental health, if someone’s feeling down or you know ‘get up and go and take some fresh air’, ‘take yourself out of a situation’, it all comes part of that* (…) *it’s definitely within conversations that we have with staff on a day to day basis you know yeah, mentally and physically.*”

P13 (TL2 I)

In contrast to the agents and team leaders, who provided insights in to their individual roles, senior team members offered a broad organisational perspective, focusing on the “*duty of care*” (P24 ST3 I) to their employees’ health and wellbeing. Each senior team member deemed their company responsible for fostering a workplace environment that enables agents to move more and sit less.

“*I think we* (company 1) *should encourage it. I think we should at least make people aware of the things that they can do, and perhaps that’s something we maybe, we don’t talk enough about or promote that we do allow people to go and make themselves a brew and have a walk or if they want to go downstairs then because that is quite a thing to promote and talk about that people can do that kind of thing in the workplace.*”

P18 (ST1 FG)

Interestingly, though, team leaders and senior team members identified a conflict between promoting agent health and wellbeing, and, business needs including meeting targets, remaining productive and adhering to personal time allocations for comfort and scheduled breaks, which were all continuously monitored

“*…we have reports which effectively tell us at any given moment of the day, the status of an agent’s phone.*”

P26 (ST3 FG)

“*Am I trying to achieve something that I can turn round to my bosses and go ’look, we improved performance’, or am I actually trying to turn round and say ’people are happier’? You’ve got to prioritise.*”

P26 (ST3 FG)

### 3.2. Interpersonal Factors

#### 3.2.1. Call Agents’ Perception of the Working Culture

Within discussions on sitting and PA at work, agents identified the concept of a working culture within each company, which they described as typically sedentary in nature in office spaces and break out areas. Agents therefore identified the working culture as a factor influencing their SB and PA at work.

“*…we’re in the culture that we are in where we are just quite happy to sit at our desks* (…) *There needs to be a bit of enthusiasm led by the business.*”

P35 (AG4 FG)

Agents commonly felt that any break in addition to scheduled break times, for example to attend a meeting, complete a working task or take a comfort break, would be judged negatively by others in the current working culture. When specifically discussing PA promotion and SB reduction, most agents and some team leaders were conscious that fellow agents and team leaders would perceive agents as unproductive, strange or disruptive if they frequently broke up their sitting.

“*…if everyone was doing it, everybody else would just go ‘look at them skiving again going outside’, and ‘how many calls have you took today?’*”

P9 (AG 1 FG)

“*…some staff probably feel as though, ‘if I go away from my desk, they’re* (team leaders) *going to think I’m skiving or not doing my work’.*”

P2 (TL1 FG)

#### 3.2.2. The Integral Role of Team Leaders

Agents, team leaders and senior team members considered team leaders integral in influencing agents PA and SB at work. Team leaders were identified as the “*key levers to pull*” P27 (ST3 FG) for initiating a shift in culture towards more active and less sedentary call agents. Agents were aware that team leaders were prone to skipping break times and eating at their desks or during meetings, yet felt that team leaders were able to influence agents’ PA and SB at work by visibly demonstrating the target behaviours within their own working practices.

“*So if all team managers and whoever and it came from top down and led by example; so if there all acting a certain way, it becomes the culture within the contact centre and then subconsciously you are going to be more active because you’re not realising it. You’re not making a conscious effort to a certain point because everybody is doing it and then it becomes normal.*”

P35 (AG4 FG)

Some team leaders and senior team members were also aware that their own behaviour at work could influence call agent behaviour and the culture surrounding PA and SB.

“*So everybody does need to have a break so that they come back fresh, but what we do is, we just slog through it, because everybody just does now,* (…) *our leaders do that as well. I’m not saying it’s their fault, it’s absolutely not their fault, but no one else will change until they see them* (team leaders) *actually changing first.*”

P25 (ST 3 FG)

## 3.3. Environmental Factors

### 3.3.1. Ergonomic Set-Up

Agents, team leaders and senior team members identified the call agents’ physical connection to their workstation, via a headset, as the main environmental barrier preventing agents from sitting less at work. This connection is essential for accurately inputting customer information onto the computer systems during calls, and ultimately maintaining productivity.

“*You can’t leave. You’ve got to capture the information whilst you’re on the phone. It’s got to be correct and accurate. So I think that’s a major barrier.*”

P36 (AG4 FG)

Agents and team leaders considered the potential impact of standing while on calls in the current ergonomic environment, and perceived poor posture and prolonged static standing as key barriers to this, and a potential musculoskeletal health hazard.

“*The headset, you know, you’d be bending over again and again. It’s not ideal. The best way to actually do the job I do, is actually being sat down.*”

P38 (AG4 FG)

### 3.3.2. Proposed Environmental Strategies

Agents, team leaders and senior team members identified height-adjustable workstations as a tangible solution to enable agents to break up their sitting time while maintaining productivity.

“*I’d like to see us* (…) *come up with a, not radical but something different like we might have banks of desks that you know that are permanently up or different ways that they can sit, or when they’ve been sat for a few hours and think ‘I’m going to move over to this hot desk and I’m going to work here because I can stand.*”

P12 (ST2 FG)

Uniquely however, senior team members acknowledged the cost associated with implementing height-adjustable workstations as a potential organisational barrier for company-wide implementation.

“*I can absolutely see the benefits of them* (height-adjustable workstations) *I really, truly can it’s just thinking about how you practically do it within this kind of working environment isn’t it, without incurring a massive cost.*”

P18 (ST1 FG)

One agent also articulated how implementing height-adjustable workstations alone may not elicit sustained changes in sitting behaviour over time.

“*So it’s just a standing up desk? I probably wouldn’t. I think that’s more due to a bit of laziness, or it’s, what can I say? I think it’d be more the novelty of it might wear off after a certain amount of time, and then I’d probably think ‘I want to sit down now’.*”

P3 (AG3 FG)

Subsequent discussions with team leaders and senior members identified alternative strategies to reduce workplace SB and address the environmental restriction preventing agents breaking up SB at work. Strategies included prompting walking, standing or active one-to-one and team meetings, short frequent breaks, prompts to break sitting time, wireless headsets, initiating more discussions about general health and wellbeing with agents, and, demonstrating buy-in to a PA and SB initiative through their own working practices.

“*You don’t have to do your 1:1 sat in a room do you? You could go for a walk.*”

P20 (TL3 FG)

“*We* (team leaders) *could promote it, walking up the stairs let’s have a challenge for the week, whoever does the most steps, get in stepometers or something.*”

P13 (TL2 I)

Agents and team leaders, however, questioned the acceptability of implementing a hot desk system of height adjustable workstations, and the feasibility of portable equipment.

“(In relation to using a hot desk) *the IT never works somewhere else, and you get your own desk with your stuff, your information and everything. You can’t transport all that around.*”

P16 (AG2 FG)

In addition, team leaders and senior staff expressed reservations to implementing short-frequent breaks due to the potential negative impact on agents’ adherence to break times and, in turn, productivity. Many agents also perceived a short break (e.g., 5 min) as inadequate.

“(Regarding short frequent breaks) *there’s more opportunity to not be adhering to it.*”

P26 (ST3 FG)

“*I mean, five minutes. What would you do in five minutes? You may as well just stand up at your desk and then sit back down again.*”

P28 (AG3 FG)

Considering call volumes in each centre were susceptible to large and sporadic fluctuations, agents and team leaders believed it was important to integrate SB reduction and PA promotion strategies into daily working practices. Senior team members had a similar perception that it would improve engagement in, and the sustainability of, health initiatives, while minimising disruption to productivity.

“*I think it’s trying to build it into your job* (…) *I like standing up or moving around a bit and twisting, so you can do that on the phone, but it’s how to do all the typing things and other things as well.*”

P8 (AG 1 FG)

“*… also so that it (reducing sitting) can be done no matter whether it’s busy or it’s quiet, because again, I think we too easily take on ideas when it’s really easy to do it, but if it becomes harder, obviously they go out the window* (…). *If we hit red alert, it means we’ve got two to three calls queuing, then everything is pulled.*”

P32 (TL3 FG)

## 3.4. Organisational Factors

### 3.4.1. Senior Team Leaders’ Perspective on Increasing Organisational Buy-In

All senior team members offered a unique insight into the organisational motives for promoting PA and reducing SB at work among agents. Motives consistently included reducing sickness absence, improving tenure and optimising productivity, with an underlying ethos that promoting healthier lifestyles at work would mutually benefit agents and the business.

“*…if staff are healthier and happier then they’re going to be more engaged within the workplace.*”

(P18 ST1 FG)

All senior staff, however, indicated that in order to invest in future interventions, an evidence-based business case was essential. Senior team members described that the business case should demonstrate the impact of an intervention on business outcomes (productivity, customer service scores, average call handling times, sickness absence and employee engagement) and employee health and wellbeing. While this suggests impact measurement was not solely driven by financial gain, documenting clear cost savings was perceived important for demonstrating a return on investment and gaining organisational investment of time or money.

“*They* (the senior team) *want to understand the usual things, why you want to do it (reduce sitting and increase PA), what the benefits for the business is going to be, the benefit to the individual, what are the risks associated if you don’t do it (reduce sitting and increase PA), a cost, and then what’s their return on investment for doing it* (reduce sitting and increase PA).”

P42 (ST4 FG)

“*I would love to be able to measure the output of the wellbeing intervention from the perspective of engagement/other business metrics such as absence* (…) *So we’re trying to make a culture change, but you can’t make a culture change without your leaders, but you also can’t make a culture change without investment, and you don’t get investment unless you can prove the benefit.*”

P26 (ST3 FG)

Measuring the impact of an intervention however highlighted a challenging paradox between delivering effective strategies to promote agents physical and psychological health, while pursuing the core business needs for return on investment.

“*…there’s an uncomfortable sort of, it’s almost a paradox to talk about ROI* (return on investment) *and wellbeing in the same breath.*”

P26 (ST3 FG)

### 3.4.2. Current Workplace Policy

Despite all four centres previously delivering PA initiatives to promote agent health and wellbeing, SB reduction and PA promotion did not appear to be defined or addressed within organisational policies or working practices.

“*We are trying to pull together a health and wellbeing strategy for* (employee), *but* (…) *I would say at the moment* (sitting time and PA) *it’s probably not defined.*”

P25 (ST3 FG)

Several current working policies within the centres, such as being allowed to eat lunch at desks, or using work computers during scheduled breaks, seemed even to discourage PA and promote SB in agents.

“*… it is very easy just to sit there when you’re having your breaks or your lunches and stay behind your computer and actually do anything.*”

P23 (TL3 FG)

Furthermore, across all centres, agents, team leaders and senior team members identified the display screen equipment assessment [37] as a prerequisite to receiving any ergonomic adaptations. Across each company, only call agents with chronic health or musculoskeletal conditions were identified as having access to approved display screen equipment strategies, including height-adjustable workstations, short and frequent breaks, and chair modifications.

“*They have the choice of standing or sitting, depending on what they want to do. We have at least two of those* (height-adjustable workstations) *sets here, but they’re for occupational health reasons only.*”

P32 (TL3 FG)

Agents acknowledged little to no organisational support in their current job role to move more and sit less. Integrating PA and SB reduction into agent and team leader roles was therefore proposed by agents as a way to enhance employee engagement and accountability to move more and sit less at work.

“*There’s no requirement within that role for us to get up and wander around. There’s nothing within the job description that says we need to do that.*”

P15 (AG2 FG)

“*…if* (promotion of active breaks and sitting reduction) *was in the team leader’s job description it might be a help.*”

P9 (AG1 FG)

Notably, an underlying theme expressed by agents and senior staff in each centre was the importance of delivering workplace initiatives in which employees felt valued and invested in.

“*…that comes down to your employees that are happy to come into work and of course with initiatives like this it’s that whole sense of the employees that they feel the companies invested in them, values them which again can of course have positive effects in terms of things we’ve kind of touched on, morale, productivity, things like that.*”

P17 (ST1 FG)

## 4. Discussion

This is the first study to explore factors influencing contact centre call agents’ workplace PA and SB from the perspective of call agents, their team leaders, and senior staff. Call agents described factors influencing their motivation to move more and sit less at work, including continuous performance monitoring, job security concerns, incivility on calls and a desire for increased autonomy over their highly controlled working practices. In contrast, team leaders and senior team members identified a conflict between promoting productivity and targets to call agents, while encouraging them to move more and sit less. Further, senior team members offered a unique insight into the organisational motives for health and wellbeing initiatives, and the importance of an evidence-based business case for future investment in PA and SB interventions. Common influential factors identified by agents, team leaders and senior team members were low knowledge and awareness of PA and SB as health-related behaviours, and PA and SB guidelines and recommendations, and, a sedentary working culture. This culture seemed compounded by perceived peer judgment, agents’ ergonomic set up and little-to-no recognition of PA promotion and SB reduction in organisational policies and job roles. In accordance with these factors, and the second study aim, strategies identified to help call agents move more and sit less at work included acknowledgement of PA and SB within policy and job roles, height-adjustable workstations, education and training sessions and greater interpersonal support.

Senior staff in the present study described that the organisational motives for implementing PA and SB strategies were to improve employee health, wellbeing and performance. Despite this, PA and SB were not acknowledged within current organisational policies or job roles. Existing practices, including being allowed to eat lunch at desks, and using work computers during scheduled breaks, appeared to discourage PA and promote SB in agents. In addition, adherence to current UK workplace legislation meant that call agents in these centres typically received remedial ergonomic support, namely to reduce existing musculoskeletal discomfort, only after a chronic medical or musculoskeletal condition was diagnosed [37]. Accordingly, current policies, practices and legislation counter recommendations for desk-based workers to stand and be active work for at least 2 h per day [43] and, organisations to have a workplace SB policy in which tasks and job roles are redesigned to include the promotion of, and opportunities for, standing and light activity [14]. Contact centres should therefore consider a preventative rather than treatment approach within their policies to support call agents’ health and wellbeing. This is especially important given that call agents accrue a greater proportion of workplace SB [26], and have higher levels of stress and depression [23], compared to other desk-based occupations.

Preventative, health promotion initiatives recalled by call agents, team leaders and senior staff focused on formal PA opportunities, with no initiatives to reduce SB identified. PA strategies identified included lunch time walking groups and exercise sessions, which have been shown effective for increasing self-reported PA among white collar workers [22]. Participants in the present study however indicated that such formal PA initiatives were not successful, due to the requirement of additional offline time, and perceived low agent motivation for the initiatives. As proposed by the participants, therefore, alternative less formal strategies embedded into current working practices, such as walking, standing or active meetings, should be considered in future contact centre trials. This approach may also help to alleviate the conflict identified by team leaders and senior team members between promoting agents’ health and wellbeing, and ensuring productivity and performance are maintained.

Similar to research in traditional office workers [17,19] and contact centre managers [28], productivity concerns and a sedentary working culture were perceived to negatively influence call agents’ PA and SB at work. The influence of these factors seems heightened in the contact centre setting, however, by the continuous objective monitoring of call agents productivity metrics and personal time, and agents’ perception that sitting less at work could reduce their productivity and hence jeopardise their job security. Equally, high daily call volumes and large, sporadic fluctuations in call volumes limit opportunities for agents to break up their sitting time. Agents consequently desired more autonomy over their working practices, which is supported by evidence that call agents have lower perceived autonomy at work than desk-based workers [23]. Accordingly, this provides further evidence to suggest that to promote physical and mental health in call agents, without influencing productivity, PA and SB strategies should be embedded within current working practices.

Another key factor negatively influencing call agents’ workplace PA and SB was their ergonomic set-up, in which agents were physically connected to their computer system via a headset. This is consistent with ergonomic restrictions identified in previous studies [17,18,19], with workers required to sit to complete computer-based tasks. Accordingly, call agents and team leaders believed the current working environment did not enable agents to reduce their workplace sitting by the recommended levels [43]. To overcome this influential factor, all stakeholders suggested height-adjustable workstations as a sitting-reduction strategy that could be embedded within current working practices.

Height-adjustable workstations were a core component of interventions reducing total and prolonged sitting in highly sedentary office-workers [12,21,47] and contact centre call agents [27,33]. Further, workplace interventions that included height-adjustable workstations observed favourable changes to HDL cholesterol [12,47] and blood glucose [53], and, maintained productivity in traditional office workers [31,47] and improved productivity in contact centre call agents [29,32]. These findings therefore suggest height-adjustable workstations have the potential to improve employee health and business metrics. Consistent with previous research, however [13,17], senior staff in the present study identified cost as a barrier to companywide implementation of height-adjustable workstations. To minimise costs, senior staff proposed a hot desk system, though agents and team leaders questioned the acceptability of this, which is somewhat supported by findings of a hot desk system not reducing sitting time in university white-collar workers [54]. Therefore, in accordance with senior team members’ perceptions in the present study and previous research [17], justifying a return on investment seems important for obtaining organisational investment in wide scale implementation of height-adjustable workstations in contact centres. Research is warranted to explore the effectiveness and the cost-effectiveness of PA and SB interventions in contact centres.

Across each contact centre, team leaders were perceived as integral for changing the prevalent sedentary working culture, by modelling target behaviours, supporting agents to break up their sitting time and instigating active working strategies. This is congruent with PA and SB trials using team leaders and managers to provide interpersonal support to traditional office workers [55,56]. Team leaders in the present study however perceived their workload as a barrier to promoting SB and PA strategies to their agents. Team leaders and senior team members subsequently suggested workplace champions as a strategy to discourage the prevalent sedentary working culture and provide agents with greater interpersonal support to move more and sit less. Workplace champions have been identified as an integral [55,56] and cost-effective [13] component in multi-component workplace PA and SB interventions in other settings, and should be considered in future contact centre trials where return on investment is vital for organisational buy-in.

Consistent with findings from a sit-stand trial in emergency call centre workers [33], call agents perceived social barriers of self-consciousness, appearing unproductive to colleagues, and disrupting colleagues, to reduce their motivation to sit less and move more at work. This is similar to findings in traditional office workers, where negative perceptions from colleagues and expectations around appropriate (i.e., sedentary) working norms were barriers to breaking up their sitting time [17]. Enhancing peer support can facilitate behaviour change [57] and appears important for promoting an activity-permissive workplace culture [18]. Moreover, consistent with findings in Flemish employees and executives [19], poor knowledge and awareness of SB and PA as health-related behaviours, and their associated guidelines and recommendations, were perceived by all stakeholder groups to contribute to the sedentary working culture and attitudes towards SB reduction and PA promotion. Knowledge enhancement is a widely recognised health behaviour change technique [57], advocated at employee and management levels [14,19]. Further, a previous multi-component intervention found education and training to be an effective component in reducing prolonged bouts of sitting and increasing PA at work in Australian office workers [58]. Research is warranted, however, to assess the efficacy of interpersonal support, and education and training sessions within the contact centre setting.

### Strengths and Challenges for Future Research

This is the first study to qualitatively explore multi-stakeholder perspectives on factors influencing contact centre call agents’ workplace PA and SB. The methodology enabled triangulation of stakeholder perspectives across multiple organisations and job roles, which reflects a more representative sample and revealed logistical, acceptability and feasibility considerations that would not have been obtained from recruiting from a single job role, contact centre or sector. Aligned to the MRC framework, this study forms the initial formative phase of a larger body of work in contact centres, and the findings will be used to tailor future interventions to the needs of stakeholders, with the hope of enhancing intervention effectiveness and sustainability [35]. The process of data collection, triangulation and data analysis followed a thematic methodology to increase the credibility and trustworthiness of the findings [41,45]. The findings are reported in line with the COREQ checklist to enhance the studies credibility and rigor [36], and were anchored to the widely recognised and adopted SEM to guide future research in this field, particularly trials [11].

Similar research in contact centres in different sectors, regions and countries may identify alternative influential factors, with research warranted to determine the generalisability of the findings. The findings and conclusions are based on cross-sectional perspectives of volunteering participants who did not participate in workplace PA and SB strategies in this study. Future trials should therefore evaluate the acceptability and feasibility of the proposed PA and SB strategies in contact centres and employees who did not participate in this study, to determine their effect on call agent behaviour and health, and business outcomes [35]. Questionnaire data are open to error from social-desirability bias and poor cognitive recall [59]; however, given the focus of the research question, self-report data was deemed appropriate to describe the sample. The number of participants per focus group was at times below the recommended 4–8; however, consistent themes emerged across the focus groups and interviews of varying sample size. The focus group schedule was not piloted; however, the schedule was grounded by existing research, current recommendations, and refined by the research team who are experienced in conducting qualitative research.

## 5. Conclusions

This original study explored multi-stakeholder perspectives of the factors influencing call agents’ workplace PA and SB across four contact centres. Agents offered insights into the impact of high occupational sitting and low PA on their physical and mental health, and factors influencing their motivation to move more and sit less at work. Team leaders, although pivotal in influencing behaviours, identified their own workload, and agents’ requirement to meet targets, as factors influencing their ability to promote agents to move more and sit less at work. Further, senior team leaders offered a broader organisational perspective on influential factors, including business needs and the importance of return on investment from PA and SB interventions.

While many factors influencing call agents’ workplace PA and SB were consistent with those in traditional office workers, unique factors, including continuous monitoring of productivity metrics and personal time, a physical connection to their workstation, and low autonomy over their working practices, seemed to limit call agents’ motivation and opportunity to move more and sit less at work. Further, participants stated that previous formal PA initiatives during or after work were unsuccessful due to the need for additional offline time and agent motivation. Accordingly, embedding a multi-level intervention into current working practices seemed important for the multiple stakeholders, while addressing productivity concerns. In accordance with the MRC framework for developing complex interventions, future research should evaluate the acceptability and feasibility of a multi-level PA and SB intervention informed by the present study to provide significant, original and robust evidence to inform organisational policy and practice in the contact centre sector.

## Figures and Tables

**Table 1:** Descriptive characteristics of contact centre employees by job role.

Characteristics	Senior Team (*n* = 12)	Team Leaders (*n* = 11)	Call Agents (*n* = 20)
Age (years)	40.3 ± 9.9	38.6 ± 12.2	41.1 ± 15.3
Female	7 (58)	5 (45)	10 (50)
White British	12 (100)	10 (91)	19 (95)
Single	3 (25)	5 (45)	15 (75)
Full-time employment	9 (75)	9 (82)	15 (75)
Tertiary education	10 (83)	5 (45)	10 (50)
Tenure (≥3 years)	6 (50)	9 (82)	3 (15)
Physical health summary (%)	52 ± 10 (28–65)	53 ± 4 (47–58)	49 ± 9 (30–65)
Mental health summary (%)	51 ± 6 (38–57)	44 ± 10 (31–50)	47 ± 10 (24–66)
Total PA (MET min·week)	869 (563)	1609 (1428)	964 (1125)
Occupational sitting time (min·day)	390.0 ± 111.4	333.0 ± 122.8	419.4 ± 57.1

Data presented as mean ± SD, or *n* (%). Total PA data is presented as median (IQR). The range of physical and mental component scores are also presented. *Note*: Data is missing for 1 agent, 1 team leader and 2 senior team members due to the participants leaving data collection after the focus group/interview but before survey completion. Each participant received an email to request the data; however, participants did not respond.

## Supplementary Materials

The following are available online at http://www.mdpi.com/1660-4601/15/7/1484/s1, Table S1: Focus group schedule.
