# Supplementary material for: Multi-Stakeholder Perspectives of Factors That Influence Contact Centre Call Agents’ Workplace Physical Activity and Sedentary Behaviour
Source: Int J Environ Res Public Health. 2018 Jul 13;15(7):1484. doi: 10.3390/ijerph15071484 (PMC6069165; doi:10.3390/ijerph15071484)
Supplement: Supplementary file 1 [file ijerph-15-01484-s001.zip › ijerph-327652-supplementary.pdf]

Table S1. Focus group schedule.

<table>
<tr><th>Rationale</th><th>Research Question</th><th>Main Question</th><th>Additional Questions/Probes</th></tr>
<tr><td rowspan="3">Linked to the MRC framework for developing complex interventions [35] it is important to establish an understanding of the environment and context of the research setting before implementing an intervention.

In order to meet Buckley [44] recommendations for achieving 2-4 hours or light activity or standing during the working day it is important to firstly understand the composition of the typical working day. This gives an insight into current working practices which maybe employed within the contact centres and may also highlight areas where PA and SB can be tackled in future initiatives.</td><td rowspan="3">Current working practices- Establishing an insight into current working practices and organisational needs and motivations for promoting sitting less and moving more.</td><td>Icebreaker

Agents- Can you describe a typical working day?

Team leaders- can you describe a call agents typical working?</td><td>Agents and team leaders- Include transport to and from work.
What is a typical work day structure?
What are your typical work day tasks/targets?
Is there any variation in your daily working tasks?
- Do you have your own desk?
- At what points in the day do you typically break your seated time?
How frequently do you have breaks?
Are they set or do you choose when they are?
Do you have to clock in or out during breaks?
Where do spend your breaks?
- What do you typically do?
Do you have regular meetings?
Team/ individual?
Where are they held?
How long do they typically last?
Where is your office located within the building?
- How do you get to your office? Stairs/lift?</td></tr>
<tr><td>Icebreaker

Senior team leaders- As an organisation what initially interested you in taking part in this research?</td><td>What does your organisation envisage for a successful initiative for health and wellbeing?</td></tr>
<tr><td>What does a healthy lifestyle mean to you?</td><td>What aspects of health are most important to you?
What is physical activity to you?
What is sedentary behaviour to you?</td></tr>
</table>

Business Need: The cost of ill physical and mental health to individual companies and to society in general is great. Annually, millions of days are lost through absenteeism and presenteeism in the workplace therefore tackling mental and physical health is mutually beneficial to both employers and employees.<br><br>Research need: Regardless of how physically active an individual is outside of work the deleterious effects of excessive sedentary time can have a negative impact on upon health [63]. Up to 90% of the working day in call centres is seated and sedentary, therefore provide key environments to target physical activity and sedentary behaviours.	Current practices (initiatives and interventions to promote PA and reduce sedentary behaviour in the workplace)	Currently what practices/ initiatives/ strategies are employed within your workplace which;<br>a)Support you / your employees to move more and<br>b) Encourage you / your employees to sit less?<br><br>2. Is there any way you think these can be improved?<br><br>3.What are the current targets/expectations for call agents in your workplace?	How is it delivered?<br>How frequently are they delivered?<br>One off/ongoing?<br>Inside or outside of working hours?<br>Where are they delivered?<br>In the office<br>Who can attend/engage?<br>Is there a booking system/drop in's/limited numbers<br>Is it compulsory?<br>How are they promoted?<br>Marketing/Advertising strategies<br>Who instigates these initiatives?<br>Organisation/Management/Employees<br>Are they any incentives for staff to increase/improve their PA?<br>Would you engage in it without an incentive<br>Could the delivery/design or the initiative be improved in any way?<br>-How is/ was this communicated to staff?

When considering the socio-ecological model [61] there may be a number of contributing factors which promote or negate PA and SB in the contact centres.<br>Exploring the barriers and facilitators factors which will enable the development of tailored future interventions which are specific to employee and employer needs.	Barriers and facilitators towards engagement- exploring employee motivation and engagement.	1. Can you identify any factors that you experience or perceive within the workplace which prevents you/your agents from breaking your sedentary time?<br><br>2. Can you identify any factors that would encourage you / your agents to break up sedentary time during the working day?<br><br>3. What would motivate you / your agents to sign up to a workplace health and wellbeing initiative?<br>Team leaders and Senior team members- In your role (team leader,HR, Manager, Director) do you face any challenges when trying to promote physical activity or to implement a new initiative?	Why do you think they are barriers towards engaging in PA or workplace initiatives?<br>2.What could encourage you to sit less during work?<br>What is the physical office environment like?<br>3.In an ideal world, what would you like to see implemented to improve your health and wellbeing at work?<br>What would you personally like to engage with?<br>Timing, type of activity, incentive, commitment, cost, location.

Business need: NICE workplace health management guidelines [64], recommend that organisations should take responsibility for fostering workplace relationships, environments and practices which address health and wellbeing throughout all workplace practices to positively impact on health outcomes and productivity.<br><br>Research Need: Buckley et al. [44] Recommends that adults should aim to accumulate 2- 4 hours of standing or light activity during the working day in order to positively impact their health and to reduce their long term risks of developing chronic illnesses.<br>Currently initiatives and interventions to reduce this have led to a reduction in total sitting time however have not yet met the recommendations.	Roles and responsibilities- What are the roles and responsibilities within contact centres with regards to promotion of PA in the workplace?	1.Within your workplace who's role and responsibility is it to promote physical activity and health?	1. Within your workplace who's role and responsibility is it to promote physical activity and health?<br>- What do you perceive your role to be moving more and sitting less at work?<br>- What do you perceive the role of your supervisor to be to help staff to move more and sit less?<br>-What would motivate you to sit less and move more?<br>-What do you think the motives are for your organisation to enable staff to move more and sit less during work?<br>Should PA/SB and health promotion be the responsibility of the individual, the organisation or both?<br>2. How often do you have supervisions?<br>-Is health and WB discussed during personal reviews/supervisions / Return to work interviews?
Buckley et al., (2015) recommendation	Establishing knowledge and awareness of PA guidelines and SB workplace recommendations	1.Do you know what the workplace recommendations are for PA and SB in the workplace?<br><br>2. Do you know what the national PA guidelines are?	1. What is your first reaction to hearing the SB (Buckley 2015) recommendations?<br>-Typically how much on average do you think you achieve per day?<br>Do you think they are achievable within your workplace?<br>– Why?<br>- If you were asked to stand or do light activities like walking for 2-4 hours during your working day how you would feel?<br>What impact do you think moving more and sitting less could have on you/ your agents?
